# Functional Alterations in the Posterior Insula and Cerebellum in Migraine Without Aura: A Resting-State MRI Study

**DOI:** 10.3389/fnbeh.2020.567588

**Published:** 2020-10-06

**Authors:** Jun Ke, Yang Yu, Xiaodong Zhang, Yunyan Su, Ximing Wang, Su Hu, Hui Dai, Chunhong Hu, Hongru Zhao, Lingling Dai

**Affiliations:** ^1^Department of Radiology, the First Affiliated Hospital of Soochow University, Soochow, China; ^2^Institute of Medical Imaging, Soochow University, Soochow, China; ^3^Department of Radiology, Tianjin First Central Hospital, Tianjin, China; ^4^Department of Neurology, the First Affiliated Hospital of Soochow University, Soochow, China

**Keywords:** migraine, functional connectivity, resting-state, degree centrality, functional magnetic resonance imaging

## Abstract

**Background**: Hypothesis-driven functional connectivity (FC) analyses have revealed abnormal functional interaction of regions or networks involved in pain processing in episodic migraine patients. We aimed to investigate the resting-state FC patterns in episodic migraine by combining data-driven voxel-wise degree centrality (DC) calculation and seed-based FC analysis.

**Methods**: Thirty-nine patients suffering from episodic migraine without aura and 35 healthy controls underwent clinical assessment and functional MRI. DC was analyzed voxel-wise and compared between groups, and FC of regions with DC differences were further examined using a seed-based approach.

**Results**: Compared with the control group, the migraine group showed increased and decreased DC in the right posterior insula and left crus I, respectively. Seed-based FC analyses revealed that migraine patients demonstrated increased right posterior insula connections with the postcentral gyrus, supplementary motor area/paracentral lobule, fusiform gyrus and temporal pole. The left crus I showed decreased FC with regions of the default mode network (DMN), including the medial prefrontal cortex (mPFC), angular gyrus, medial and lateral temporal cortex in patients with migraine. Furthermore, pain intensity positively correlated with DC in the right amygdala/parahippocampal gyrus, and migraine frequency negatively correlated with FC between the left crus I and mPFC.

**Conclusion**: Patients with episodic migraine without aura have increased FC with the right posterior insula and decreased FC within the DMN, which may underlie disturbed sensory integration and cognitive processing of pain. The left crus I-mPFC connectivity may be a useful biomarker for assessing migraine frequency.

## Introduction

Migraine is a common and debilitating episodic neurological disorder with a 1-year prevalence of about 12% in the general population (Lipton et al., 2007). It is characterized by recurrent, unilateral, moderate or severe, throbbing, and pulsating headaches, often accompanied by nausea, vomiting, and hypersensitivities to visual, auditory, olfactory, and somatosensory stimuli (Schwedt, 2013). This disabling disease causes significant limitations in daily life with effects on emotional behavior and relational aspects, as well as cognitive functions (Santangelo et al., 2015). So far, however, the pathophysiology of migraine remains incompletely understood.

During the past decade, advanced neuroimaging modalities are increasingly used to examine the underlying mechanisms of migraine. Mounting evidence suggests that migraine arises from a primary brain dysfunction involving a complex neuronal network (Sprenger and Borsook, 2012; Messina et al., 2018). Resting-state functional connectivity (FC) MRI is a powerful method to investigate functional coupling of specific brain areas and functional networks. Thus, a large number of migraine studies have utilized the resting-state imaging technique and the results have indicated atypical connectivity between regions involved in pain processing (Schwedt et al., 2015; Messina et al., 2018). Brain regions shown to have altered FC in patients with migraine include those that participate in sensory-discriminative processing of pain (e.g., the somatosensory cortex and posterior insula regions), affective processing (e.g., the anterior insula and amygdala), cognitive processing (e.g., the prefrontal cortex and hippocampus), and pain modulation (e.g., the periaqueductal gray; Schwedt and Chong, 2015). In addition, several resting-state networks, such as the salience network, default mode network (DMN), and executive network, have been reported to exhibit altered FC in migraine patients (Schwedt and Chong, 2015; Schwedt et al., 2015).

However, findings across the resting-state MRI studies on migraine are complex and often inconsistent (Skorobogatykh et al., 2019). For example, while some researchers reported increased FC for the insula (Hadjikhani et al., 2013; Yuan et al., 2013) or the periaqueductal gray (Mainero et al., 2011) in patients with migraine, others reported decreased FC in these regions (Chen et al., 2017; Yu et al., 2017b; Soheili-Nezhad et al., 2019). Furthermore, though reduced FC within the DMN and executive network has been repeatedly detected, negative or opposite results have also been observed in some migraine studies (Ellingson et al., 2019). The discrepant findings may be due to the small sample size, patients’ clinical heterogeneity, and the varied methodological approach of data analysis (Skorobogatykh et al., 2019). Indeed, while some FC studies on migraine used seed-based methods, others utilized independent component analysis, making it hard to compare results across studies. It is noteworthy that most of these MRI studies focused on predefined brain areas or resting-state networks and thus were unable to examine the whole-brain functional features. It is posited that the use of analyses based on the region of interest, rather than whole-brain analyses, is a substantial limitation of current functional MRI studies (Schwedt et al., 2015).

In this resting-state MRI study, we conducted a data-driven voxel-wise degree centrality (DC) analysis to investigate the network property over the whole brain in episodic migraine without aura. Voxel-wise DC is a graph-based index that represents the number of direct connections for a given voxel with the rest of the whole brain (Buckner et al., 2009; Lohmann et al., 2010). This measure could reflect the impact and significance of brain hubs in network information communication and has been proved to have high sensitivity, specificity, and test-reliability (Zuo and Xing, 2014). On the basis of the voxel-wise DC results, seed-based FC analysis was then performed to determine connections that contributed to the DC differences between patients with migraine and healthy controls. We hypothesized that patients with migraine would present DC and FC alterations in regions associated with pain processing, such as the insula and somatosensory cortex, and some changes may correlate with disease severity reflected by migraine frequency and headache intensity.

## Materials and Methods

### Participants and Clinical Assessment

We enrolled thirty-nine right-handed patients with episodic migraine without aura from the Department of Neurology of the first affiliated hospital of Soochow University between December 2016 and September 2017. The diagnosis of episodic migraine without aura was made by a senior neurologist investigator according to the international classification of headache disorders, 3rd edition, beta version (ICHD-3 beta; Olesen et al., 2013). Migraine patients had not experienced migraine attacks for at least 3 days prior to and 1 day after MRI scanning. To avoid the effects of pharmacologic treatment, no prophylactic treatments were allowed during the last 3 months. The control group consisted of 35 right-handed healthy individuals. Healthy controls were recruited by advertisement and had no history of any primary headache disorders or other types of headache. A family history of no migraine or psychiatric disorders was also required for the healthy control group.

General exclusion criteria included age <18 years or >65 years; left-handedness, migraine with aura; a headache attack during an MRI scan or within 24 h after scanning; cardiovascular disease or trauma; metabolic disorders such as diabetes mellitus; any other neurological or psychiatric disease; other pain conditions; drug or alcohol abuse; pregnancy or breastfeeding; MRI contraindications (i.e., claustrophobia and ferromagnetic implant) and excessive movement during MRI scanning (translation >1.5 mm or rotation >1.5° at any direction). The recorded clinical data of patients included migraine attack frequency (number of migraine days per month) and pain intensity, which was rated with a 10-point visual analog scale (VAS) from 0 (none) to 10 (very severe). The study was conducted in accordance with the declaration of Helsinki and approved by the ethics committee of the first affiliated hospital of Soochow University. Written informed consent was obtained from all participants.

### MRI Data Acquisition

MRI scans were performed using a 3.0 Tesla scanning system (MAGNETOM Skyra, Siemens Healthcare, Erlangen, Germany) at the department of radiology, the first affiliated hospital of Soochow University. Earplugs and tight padded clamps were used to minimize noise exposure and head motion. The axial resting-state functional images were acquired using an echo-planar imaging sequence with the following parameters: TR/TE = 2,000/30 ms, flip angle = 90°, FOV = 256 × 256 mm^2^, matrix = 64 × 64, slice number = 33, slice thickness/gap = 4/0 mm, total volume number = 240. The axial sections were placed approximately parallel to the anterior commissure-posterior commissure line. High-resolution T1-weighted images were obtained using a sagittal fast spoiled gradient recalled echo sequence using the following parameters: TR/TE = 2,300/2.98 ms, matrix = 256 × 256, FOV = 256 × 256 mm^2^, slice thickness = 1 mm. Scanning was terminated if the participant complained of any discomfort. During the resting-state scan, participants were instructed to lie still with their eyes closed, not to fall asleep or think about anything in particular.

### Data Preprocessing

The functional images were preprocessed with Statistical Parametric Mapping (SPM12) software package^1^ and Resting-State fMRI Data Analysis Toolkit (REST^2^). The first 10 volumes were discarded to reduce the effect of instability magnetization at the beginning of resting-state scans. The remaining functional images were slice-time corrected, realigned, and co-registered with the individual T1-weighted images. The co-registered anatomical images were then segmented into gray matter (GM), white matter (WM), and cerebrospinal fluid (CSF), and spatially normalized into standard Montreal Neurological Institute (MNI) space with a final size of 3 × 3 × 3 mm^3^. The resulting normalization matrix was then applied to the functional images. The functional data were further detrended to correct for general signal drift and band-pass filtered (0.01–0.08 Hz) to reduce low-frequency drift and high-frequency noise. Finally, nine nuisance covariates, including the average time series for global signal, WM, CSF and six motion parameters were sequentially regressed from the time series. Notably, in seed-based FC analysis, spatial smoothing followed spatial normalization with an 8-mm full width at a half-maximum Gaussian kernel, while it was conducted after DC calculation in the voxel-wise DC analysis.

### DC and Seed-Based FC Analysis

Voxel-wise DC analysis was performed with the REST software. For each subject, preprocessed functional data were subjected to voxel-based whole-brain correlation analysis, resulting in a Pearson correlation coefficient (*r*) matrix. DC is defined as the sum of weights (*r*-values) of significant functional connections (*r* > 0.25) for each voxel (Zuo and Xing, 2014). In the present study, we did not involve negative connections in the DC calculation, in keeping with typical graph analyses of weighted networks (Power et al., 2010). For further statistic analyses, the individual DC maps were converted to *z*-value maps, which were then spatially smoothed with an 8-mm full width at a half-maximum Gaussian kernel.

To explore which connections contributed to altered DC in patients with migraine, seed-based FC analyses were further conducted with regions showing significant between-group differences as regions of interest (ROIs). Specifically, for each individual, mean time series of each seed-point were calculated by averaging the functional MRI time series for all voxels within each ROI and then correlated with time series of the rest of the whole brain in a voxel-wise way using the preprocessed functional images. The resultant correlation maps were subsequently normalized with Fisher’s r to Z transformation.

### Statistical Analysis

The demographic and clinical data were analyzed using SPSS statistical analysis software (version 16.0, SPSS Inc., Chicago, IL, USA). Gender distribution was tested for statistically significant between-group differences with the chi-square test, while the continuous data (age, education level) were tested with independent two-tailed unpaired Student’s *t*-test. Statistical significance was set at an alpha level of 0.05.

For DC and seed-based FC maps, random-effect one-sample *t*-tests were performed in SPM12 to depict the DC distribution pattern and which brain areas showed significant connections with the seed region in each group, respectively, with a false discovery rate corrected threshold of *p* < 0.05. Between-group differences in DC and seed-based FC were analyzed using random-effect two-sample *t*-tests in SPM12 with age, gender, and education level as covariates.

Moreover, in the migraine group, multivariate regression analyses were conducted to explore the association between clinical indices (migraine frequency, pain intensity) and brain measures (DC, seed-based FC) using SPM12 in a voxel-wise way, with age, gender, and education level as covariates. For both two-sample *t*-tests and multivariate regression analyses, a threshold adjustment based on Monte Carlo simulations was applied to correct for multiple comparison using the AlphaSim program within REST software. Significant clusters were identified with a combined height-extent threshold (voxel-wise *p* < 0.001 and cluster size > 23), corresponding to a false-positive rate of *p* < 0.05.

## Results

Demographic and clinical characteristics of the study population are provided in Table 1. There were no significant differences between the migraine and control groups in gender distribution, age and level of education (*p* > 0.05). The episodic migraine group had an average migraine frequency of 3.75 ± 2.64 days per month, and a mean pain intensity of 6.22 ± 1.77.

**Table 1:** Demographic and clinical characteristics of patients and healthy controls.

	EM (*n* = 39)	HC (*n* = 35)	*p*-value
Gender (males/females)	9/30	15/20	0.070^a^
Age (year)	39.74 (11.59)	34.91 (10.89)	0.070^b^
Education (year)	10.33 (4.02)	11.80 (4.92)	0.171^b^
Migraine frequency	3.75 (2.64)
Pain severity	6.22 (1.77)

*^a^*P*-value obtained with Chi-square test; ^b^*p*-value obtained with independent *t*-test. Continuous variables are given as mean (standard deviation). EM, episodic migraine; HC, healthy control*.

**Figure 1:**
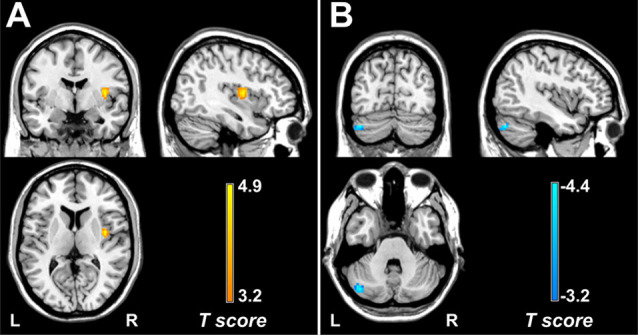
Brain regions with DC alterations in migraine. Compared with healthy controls, patients with episodic migraine without aura show significantly increased DC values in the right posterior insula **(A)** and decreased DC values in the left crus I **(B)**. DC, degree centrality.

Within each group, brain regions with relatively high DC values were bilaterally distributed in the precuneus, middle cingulate cortex, supplementary motor cortex (SMA), precentral gyrus, postcentral gyrus, and superior and middle temporal gyrus (Supplementary Figure 1). Compared with the control group, the migraine group showed increased DC value in the right posterior insula (cluster size = 35 voxels, peak *t*-score = 4.88, MNI_XYZ_ = 39, −3, and 9; Figure 1A) and decreased DC value in the left cerebellum (crus I: cluster size = 59 voxels, peak *t-score* = −4.35, MNI_XYZ_ = −42, −75 and −33; Figure 1B).

Secondary seed-based FC analyses showed that in both control and migraine groups, the right posterior insula was functionally connected with the bilateral entire insula, thalamus, basal ganglia, dorsolateral prefrontal cortex, posterior orbitofrontal cortex, inferior parietal lobule, SMA, paracentral lobule (PCL), dorsal anterior cingulate cortex, regions of the sensorimotor network, occipital cortex, and cerebellum (Supplementary Figure 2). Within each group, the left crus I showed significant connectivity with the bilateral entire cerebellum, thalamus, as well as regions of the previously described DMN (Fox et al., 2005; Buckner et al., 2008) including the precuneus, posterior cingulate cortex, medial and dorsolateral prefrontal cortex, lateral parietal lobule, and medial and lateral temporal cortex (Supplementary Figure 3).

**Figure 2:**
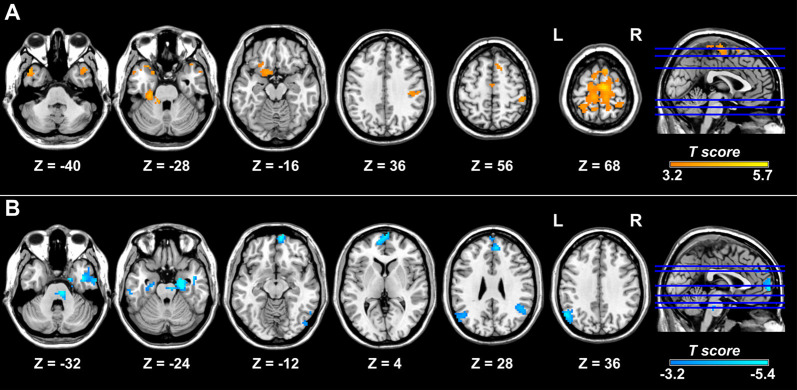
Brain regions showing altered FC with the right posterior insula and left crus I in migraine. Relative to the control group, the migraine group shows increased FC of the right posterior insula with the right postcentral gyrus, bilateral supplementary motor area, paracentral lobule, and temporal pole and left fusiform gyrus. **(A)** Patients with migraine show decreased FC between the left crus I and regions of the default mode network, including the bilateral medial prefrontal cortex, angular gyrus, and medial and lateral temporal cortex **(B)**. FC, functional connectivity.

Compared with controls, patients with migraine exhibited increased right posterior insula connectivity with the bilateral SMA/PCL, right postcentral gyrus, left orbitofrontal gyrus and fusiform gyrus, bilateral temporal pole, and cerebellum (Table 2; Figure 2A). No regions showed decreased FC with the right posterior insula in the migraine group. Furthermore, the left crus I showed decreased FC with the bilateral angular gyrus, medial prefrontal cortex (mPFC), hippocampus/parahippocampal gyrus (PHG), middle temporal gyrus/inferior temporal gyrus, left temporal pole, right cerebellum and brainstem in the migraine vs. control comparison (Table 3; Figure 2B). The migraine group showed no increased connectivity for the left crus I as compared with the control group.

**Table 2:** Brain regions showing right insula functional connectivity differences between migraine patients and healthy controls.

Brain region	Lat	Voxels	MNI coordinate			Peak *t*-score
			X	Y	Z	
SMA/PCL	R	239	9	−15	72	5.67
SMA/PCL	L	202	−6	−30	72	4.52
PoCG	R	78	51	−33	54	4.42
OFG	L	41	−29	24	−21	4.21
TP	L	39	−45	15	−36	4.42
TP	R	43	45	15	−39	3.85
Fusiform gyrus	L	26	−30	−27	−30	4.65
Cerebellum	R	161	24	−45	−54	5.46
Cerebellum	L	26	−27	−45	−57	4.06

*MNI, Montreal Neurological Institute; SMA, supplementary motor area; PCL, paracentral lobule; PoCG, postcentral gyrus; OFG, orbital frontal gyrus; TP, temporal pole; Lat, lateralization; L, left; R, right*.

**Table 3:** Brain regions showing left cerebellum functional connectivity differences between migraine patients and healthy controls.

Brain region	Lat	Voxels	MNI coordinate			Peak *t*-score
			X	Y	Z	
Angular gyrus	L	122	−54	−63	36	−4.33
Angular gyrus	R	63	45	−51	30	−3.93
mPFC	R	426	9	63	−9	−5.41
MTG/ITG	L	48	−60	−24	−21	−3.90
MTG/ITG/TP	R	134	45	0	−30	−4.62
ITG	R	36	57	−54	−18	−4.08
IOG	R	32	42	−87	−15	−3.83
HP/PHG	R	118	24	−15	−27	−5.33
HP/PHG	L	32	−24	−12	−24	−3.75
Brainstem	R	89	9	−27	−33	−4.91
Cerebellum	R	52	48	−57	−48	−4.20

*MNI, Montreal Neurological Institute; mPFC, medial prefrontal cortex; MTG, middle temporal gyrus; ITG, inferior temporal gyrus; TP, temporal pole; IOG, inferior occipital gyrus; HP, hippocampus; PHG, parahippocampal gyrus; Lat, lateralization; L, left; R, right*.

**Figure 3:**
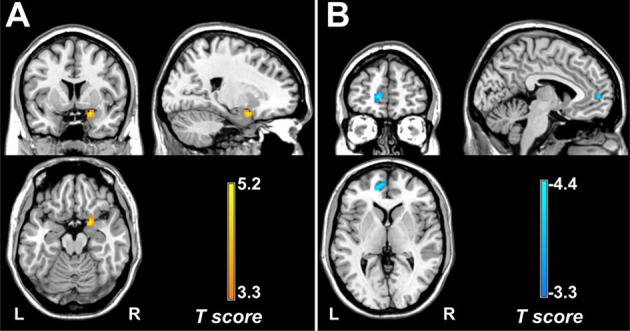
Correlation of DC and FC with clinical features. Across all patients with migraine, pain intensity positively correlates with DC values in the right amygdala/PHG. **(A)** Migraine frequency negatively correlates with FC between the left crus I and medial prefrontal cortex **(B)**. DC, degree centrality; FC, functional connectivity; PHG, parahippocampal gyrus.

Multivariate regression analyses revealed that DC values in the right amygdala/PHG (cluster size = 24 voxels, peak *t*-score = 5.23, MNI_XYZ_ = 21, 6, and −18) were positively correlated with the pain intensity in patients with migraine without aura (Figure 3A). In addition, FC between the left crus I and left mPFC (cluster size = 43 voxels, peak *t-score* = −4.88, MNI_XYZ_ = −12, 51, and 3) showed a negative correlation with migraine frequency in all patients with migraine (Figure 3B).

## Discussion

This study examined intrinsic functional architecture by analyzing voxel-vise DC in patients with episodic migraine without aura. As a data-driven and model-free approach, the centrality mapping allows us to assess the organization of large-scale functional connectivity across the entire brain connectome, without requiring selection *a priori* of nodes or networks of interest. We found increased DC in the right posterior insula and decreased DC in the left crus I in migraine, suggesting that these two regions are more and less centrally embedded in the whole brain functional connectome, respectively, in patients with this disorder. The findings fitted previous studies based on the regions of interest frequently showing altered function in the insula (Borsook et al., 2016) and also expand the foci in migraine pathophysiology to the cerebellum, a region often neglected, by showing that it may serve as a source of interference for information flow with the connectome. Our further seed-based analyses revealed that the abnormalities detected in network information processing resulted from enhanced right-posterior insula FC with multiple regions related to pain processing and reduced cerebellum FC with components of the DMN. Moreover, pain intensity was associated with increased DC in the right amygdala/PHG, and migraine frequency was associated with decreased FC between left crus I and mPFC, suggesting that altered functional interaction may reflect migraine symptom severity.

The insula is involved in emotion, homeostasis, autonomic function, sensation, salience, and awareness (Nieuwenhuys, 2012) and has been called “a multidimensional integration site for pain” (Borsook et al., 2016). Compared with the anterior insula, the posterior insula is more closely connected to the SMA, premotor, sensorimotor, and middle-posterior cingulate cortex, indicating a role for the posterior part in sensorimotor integration (Cauda et al., 2011). Our data showed that the right posterior insula had increased DC as well as increased FC with the postcentral gyrus, SMA/PCL, fusiform gyrus, and temporal pole in migraine patients relative to controls. Right-sided lateralization of insula abnormalities has also been observed in a number of structural and functional studies of pain or migraine (Symonds, 2006; Maleki et al., 2012, 2015; Zhang et al., 2017). Partially consistent with our results, the posterior insula showed reduced habituation associated with amplification of trigeminal brainstem input in a previous study on episodic migraine without aura (Lee et al., 2017). In addition, prior literature showed that female patients with migraine had thicker posterior insula relative to male patients with migraine and healthy controls of both sexes (Maleki et al., 2012). The postcentral gyrus predominately participates in sensory-discriminative pain processing, and the PCL is correlated with movement of the body in space (Rushworth et al., 2003). The SMA is involved in executive functions, such as working memory and motor control (Veltman et al., 2005; Thomaes et al., 2010). The temporal pole is an associative multisensory area, plays a role assigning affective tone to short-term memories related to pain (Zhao et al., 2013; Schwedt et al., 2015), and has been shown to be hyper-excitable in migraine patients (Moulton et al., 2011). The fusiform gyrus is known to be involved in cognitive pain processing (Glass et al., 2011; Mehnert and May, 2019) and has been reported to be hyperactive in episodic migraine (Schwedt et al., 2014). Overall, enhanced connectivity between the right posterior insula and these regions may be related to atypical multisensory processing, impaired pain appraisal, and lead to sensory hypersensitivity, and greater vigilance and attention to pain in migraine.

In the present study, the left crus I showed significant connectivity with regions of the DMN, brainstem, and thalamus in both migraine and control groups. Supporting our findings, Krienen et al revealed a great contribution of the crus I to the DMN in a resting-state study on segregated fronto-cerebellar circuits (Krienen and Buckner, 2009). In the migraine vs. control comparison, we found decreased left crus I connectivity mainly with key nodes of the DMN, including the bilateral mPFC, angular gyrus, hippocampus/PHG, and lateral temporal cortex. Though the role of the cerebellum (including crus I) in migraine is not well defined, it is known to exert an inhibitory control on cerebral cortex (Brighina et al., 2009; Moulton et al., 2010; Mehnert and May, 2019). The DMN is involved in several cognitive processes, such as memory, problem solving, and planning (Buckner et al., 2008; Lo Buono et al., 2017), as well as perception and processing of painful stimuli (Soheili-Nezhad et al., 2019). Specifically, the hippocampus and PHG are associated with learning and memory formation, as well as pain-related attention and anxiety (Buckner et al., 2008; Liu and Chen, 2009). The lateral temporal cortex is linked to semantic processing and memory retrieval (Buckner et al., 2008). The angular gyrus is considered a connector hub for global integration of information (de Pasquale et al., 2012). The mPFC participates in self-referential processing (Legrand and Ruby, 2009) and is also thought to mediate attenuation of pain perception *via* cognitive control mechanisms (Wiech et al., 2008). Partially in accordance with our results, previous research showed structural alteration in the left crus I (Granziera et al., 2013; Mehnert and May, 2019; Liu et al., 2020) and mPFC (Soheili-Nezhad et al., 2019) in episodic migraine without aura. Amin *et al* found decreased right cerebellum (crus I) connectivity with the DMN during induced migraine attacks in migraine patients (Amin et al., 2016). With regard to resting-state MRI, reduced FC of the DMN has been repeatedly observed in multiple studies (Tessitore et al., 2013; Yu et al., 2017a). Given the cerebellum’s dominantly inhibitory role in pain modulation, the crus I’s contribution to DMN, and the involvement of DMN in cognitive processes, we speculated that reduced FC between the left crus I and classic regions of the DMN may reflect altered organization of this resting-state network, leading to disturbed physiological mechanism to cognitively attenuate pain perception.

Multivariate regression analyses revealed a positive correlation between pain intensity and DC value in the right amygdala/PHG. The amygdala is well known for its ability to activate arousal systems that affect information processing across the brain (Ledoux and Brown, 2017). It is the right amygdala that plays a major role in the processing and emotional component of pain (Carrasquillo and Gereau, 2008). Thus, greater DC value in the right amygdala in migraine patients with higher pain intensity may indicate amplification of nociception and increased emotional responses. In line with this view, a recent positron emission tomography study showed that lower μ-opioid receptor non-displaceable binding potential in the right amygdala greatly correlated with the severity of migraine attack and, to some extent, the decrease in the ictal thermal pain threshold in patients with chronic migraine (Jassar et al., 2019). Furthermore, we found a negative correlation between migraine frequency and FC between the left crus I and mPFC. Consistent with our data, Kim et al. demonstrated that gray matter volume changes in the mPFC negatively correlated with headache duration and lifetime headache frequency (Kim et al., 2008). Additionally, several studies pointed out that dysregulated FC of the mPFC with other brain regions in the DMN was related to chronic pain patients’ degree of rumination and intensity about their pain (Loggia et al., 2013).

Several limitations of the current study should be noted. First, the results should be considered preliminary given the relatively small sample size. Second, the matching for gender, age, and education was not well balanced and may compromise the results, though these characteristics had no statistically significant between-group differences. Third, it was unable to be established whether the detected FC alterations were specific to migraine without aura or represent common changes in chronic pain of other different types. In fact, reduced FC within the DMN has also been observed in other chronic pain disorders, including osteoarthritis and low back pain (Loggia et al., 2013; Kucyi et al., 2014). Fourth, the cross-sectional nature of this study did not allow us to ascertain the causal relationship between the brain function abnormality and migraine, i.e., whether FC alterations predispose a person to migraine or result from recurrent migraine attacks.

## Conclusion

In summary, this study demonstrated increased FC between the right posterior insula and brain areas involved in sensory and cognitive processing of pain, as well as decreased FC of the left crus I with regions belonging to the DMN in patients with migraine without aura. Stronger FC of the posterior insula may underlie atypical sensory integration and hyper-vigilance for pain. Lower FC within the DMN may be related to disturbed inhibition of pain *via* cognitive control mechanisms. The connectivity between the left crus I and mPFC may be a useful biomarker for assessing migraine severity.

## Data Availability Statement

The raw data supporting the conclusions of this article will be made available by the authors, without undue reservation.

## Ethics Statement

The studies involving human participants were reviewed and approved by the ethics committee of the first affiliated hospital of Soochow university. The patients/participants provided their written informed consent to participate in this study.

## Author Contributions

LD and HZ made substantial contributions to the interpretation of data as well as in drafting the manuscript. JK and YY conducted data processing and statistical analysis and wrote the first draft of the manuscript. XZ and YS made a contribution to the interpretation of data. XW and SH were involved in recording data. HD and CH gave critical revision of the manuscript for important intellectual content. All authors contributed to the article and approved the submitted version.

## Conflict of Interest

The authors declare that the research was conducted in the absence of any commercial or financial relationships that could be construed as a potential conflict of interest.

## Footnotes

^1^http://www.fil.ion.ucl.ac.uk/spm/

^2^http://www.restfmri.net
