# Supplementary material for: Functional Alterations in the Posterior Insula and Cerebellum in Migraine Without Aura: A Resting-State MRI Study
Source: Front Behav Neurosci. 2020 Oct 6;14:567588. doi: 10.3389/fnbeh.2020.567588 (PMC7573354; doi:10.3389/fnbeh.2020.567588)
Supplement: Supplementary file 1 [file Data_Sheet_1.DOCX]

***Supplementary Material***

**Supplementary Figures**


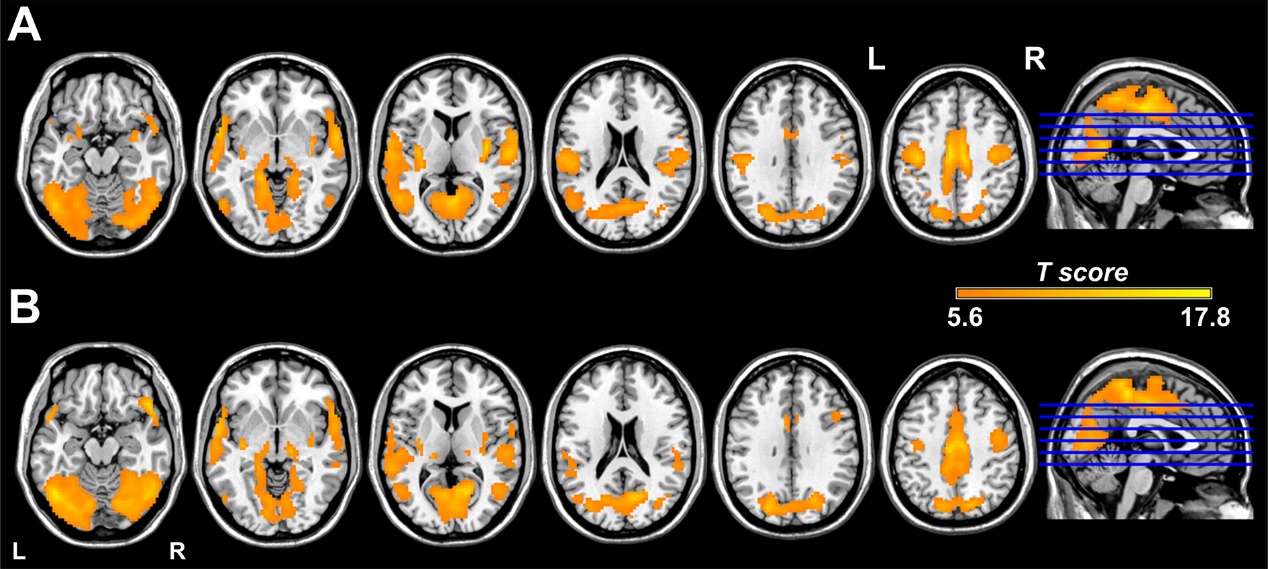


**Supplementary Figure 1.** DC maps of the migraine and control groups. Within both the migraine group (A) and the control group (B), brain regions with relatively high DC values are bilaterally distributed in the precuneus, middle cingulate cortex, supplementary motor cortex, precentral gyrus, postcentral gyrus, superior and middle temporal gyrus. DC, degree centrality.


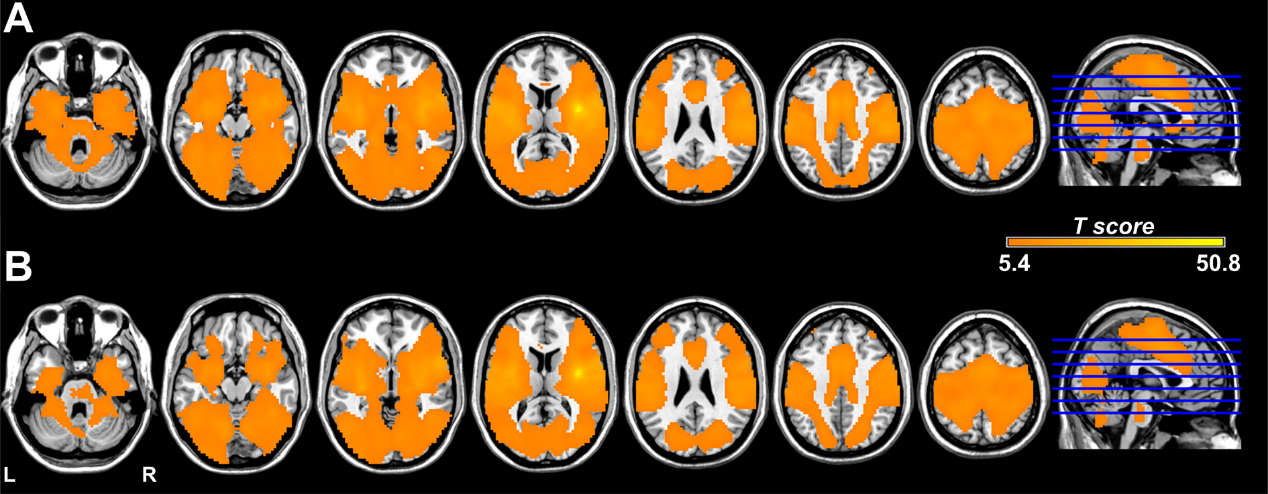


**Supplementary Figure 2.** Brain regions with significant functional connectivity with the right posterior insula. In both the migraine group (A) and the control group (B), the right posterior insula is functionally connected with the bilateral entire insula, thalamus, basal ganglia, dorsolateral prefrontal cortex, posterior orbitofrontal cortex, inferior parietal lobule, supplementary motor area, paracentral lobule, dorsal anterior cingulate cortex, regions of the sensorimotor network, occipital cortex, and cerebellum.


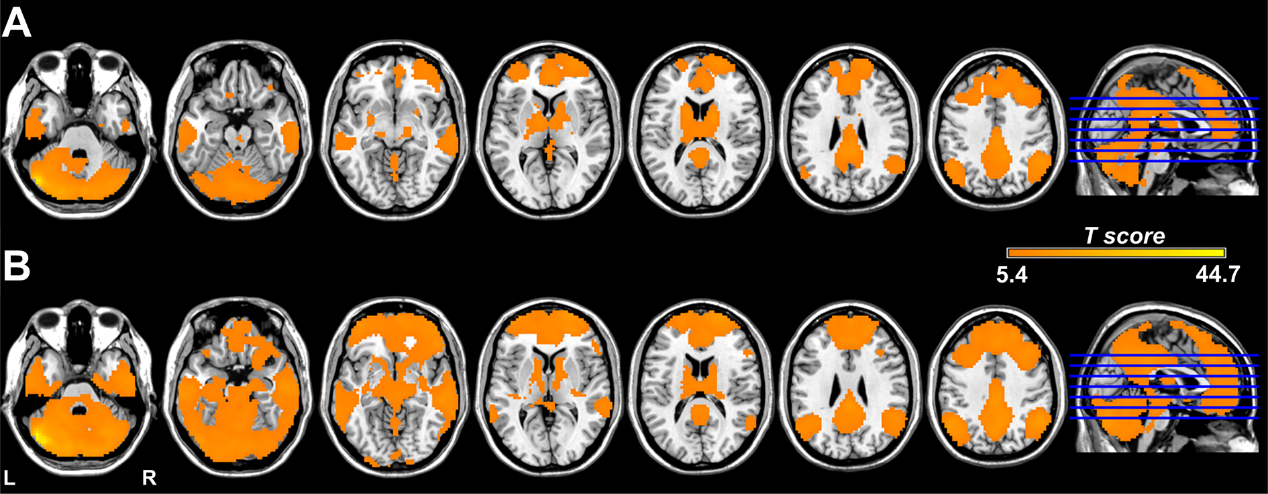


**Supplementary Figure 3.** Brain regions with significant functional connectivity with the left crus I. In both the migraine group (A) and the control group (B), the left crus I shows significant connectivity with almost the entire bilateral cerebellum, bilateral thalamus, as well as regions of the default mode network.
